# A Modular Cloning Toolkit for the production of recombinant proteins in *Leishmania tarentolae*

**DOI:** 10.15698/mic2024.04.821

**Published:** 2024-04-30

**Authors:** Katrin Hieronimus, Tabea Donauer, Jonas Klein, Bastian Hinkel, Julia Vanessa Spänle, Anna Probst, Justus Niemeyer, Salina Kibrom, Anna Maria Kiefer, Luzia Schneider, Britta Husemann, Eileen Bischoff, Sophie Möhring, Nicolas Bayer, Dorothée Klein, Adrian Engels, Benjamin Gustav Ziehmer, Julian Stieβ, Pavlo Moroka, Michael Schroda, Marcel Deponte

**Affiliations:** 1 Faculty of Biology, Molecular Biotechnology & Systems Biology, RPTU Kaiserslautern, D-67663 Kaiserslautern, Germany.; 2 Faculty of Chemistry, Comparative Biochemistry, RPTU Kaiserslautern, D-67663 Kaiserslautern, Germany.; 3 Faculty of Computer Science, RPTU Kaiserslautern, D-67663 Kaiserslautern, Germany.

**Keywords:** iGEM, Leishmania tarentolae, LEXSY expression, MoClo, recombinant protein production, SARS-CoV-2, spike protein

## Abstract

Modular Cloning (MoClo) is based on libraries of standardized genetic parts that can be directionally assembled via Golden Gate cloning in one-pot reactions into transcription units and multigene constructs. Here, a team of bachelor students established a MoClo toolkit for the protist *Leishmania tarentolae* in the frame of the international Genetically Engineered Machine (iGEM) competition. Our modular toolkit is based on a domesticated version of a commercial LEXSY expression vector and comprises 34 genetic parts encoding various affinity tags, targeting signals as well as fluorescent and luminescent proteins. We demonstrated the utility of our kit by the successful production of 16 different tagged versions of the receptor binding domain (RBD) of the SARS-CoV-2 spike protein in *L. tarentolae* liquid cultures. While highest yields of secreted recombinant RBD were obtained for GST-tagged fusion proteins 48 h post induction, C-terminal peptide tags were often degraded and resulted in lower yields of secreted RBD. Fusing secreted RBD to a synthetic *O*-glycosylation SP20 module resulted in an apparent molecular mass shift around 10 kDa. No disadvantage regarding the production of RBD was detected when the three antibiotics of the LEXSY system were omitted during the 48-h induction phase. Furthermore, the successful purification of secreted RBD from the supernatant of *L. tarentolae* liquid cultures was demonstrated in pilot experiments. In summary, we established a MoClo toolkit and exemplified its application for the production of recombinant proteins in *L. tarentolae*.

## INTRODUCTION

*Leishmania tarentolae* is a trypanosomatid parasite that was first isolated from the white-spotted wall gecko *Tarentola annularis* in 1914 and that is non-pathogenic for humans [1–3]. However, there could be transiently infectious strains based on the detection of antibodies against *L. tarentolae* and of parasite DNA in human blood samples [4]. The parasite has become a protist model organism for RNA editing [5–7], mitochondrial protein import [8, 9], and drug screening [2]. *L. tarentolae* extracts are also used for efficient cell-free protein synthesis with unpurified PCR products [10]. Furthermore, systems for recombinant protein production in *L. tarentolae* allow ease of use as in *Escherichia coli* or yeast, but also efficient eukaryotic protein folding and mammalian-type posttranslational modifications of target proteins as exemplified for phosphorylated human p53 or the large heterotrimeric glycoprotein laminin-332 [3, 11–13]. *L. tarentolae* produces mammalian-type biantennary *N*-glycans containing galactose, fucose, and mannose, which is of particular interest for the production of recombinant mammalian proteins that require specific glycans for their functionality [1, 3, 12–15]. Axenic promastigote cultures of *L. tarentolae* are grown on agar plates and in a variety of liquid media [6, 9, 16–18]. Doubling times and final cell densities of agitated liquid cultures are usually around 6-9 hours and 1-3 × 10^8^ cells/mL, respectively [6, 16, 18]. Even higher cell densities can be reached in aerated and stirred bioreactors further contributing to the attractiveness of *L. tarentolae* for research and recombinant protein production [7, 16]. The *L. tarentolae* genome is sequenced and annotated [17, 19], and a simple, fast and efficient genetic manipulation of *L. tarentolae* promastigotes has been established based on the versatile CRISPR-Cas9 LeishGEdit technology [9, 18, 20]. Translation initiation efficiency and protein abundance in *L. tarentolae* depend on the pre-ATG triplet and the coding region [21], with the codon usage generally being a key factor in trypanosomatids [22]. Available expression systems feature inducible and high-level expression from cassettes that integrate into the *L. tarentolae* genome or that are on episomally maintained plasmids (www.jenabioscience.com) [23]. These commercial systems were used, for example, to produce a recombinant human G-protein-coupled receptor [24], a rodent purine-pyrimidine permease [25], the secreted precursors of the cysteine protease legumain from human or *Arabidopsis thaliana* [26, 27], or the extracellular domains of mammalian glycoprotein VI and the receptor for advanced glycation end products [28]. However, traditional and commercial *L. tarentolae* expression systems employ classical cloning techniques, which often represent a bottleneck, for example, if the best type and position of an affinity tag needs to be tested empirically. Classical cloning techniques typically allow only two genetic parts to be combined at a time with low efficiency. This problem is solved with the Modular Cloning (MoClo) system [29].

MoClo is a synthetic biology tool utilizing standardized genetic parts and standardized part assembly routes [29, 30]. Efficient assembly of several predefined genetic parts in a single reaction is achieved by Golden Gate cloning using Type IIS restriction enzymes BsaI and BbsI as well as T4-DNA ligase [31, 32]. Standardization of the genetic parts means that they must lack internal BsaI and BbsI recognition sites and are cloned into specific, so-called level 0 vectors [29, 33, 34]. BsaI digestion of these level 0 vectors releases the genetic parts with characteristic 4-nucleotide (nt) overhangs. These represent defined fusion sites flanking the functional parts of a transcription unit: the promoter, the 5'-untranslated region (UTR), a potential signal peptide- and/or N-terminal tag-encoding sequence, the coding sequence, a potential C-terminal tag-encoding sequence, the 3'-UTR, and the terminator [29, 33, 34]. These parts are then directionally assembled into a complete transcription unit within a level 1 destination vector present in the same restriction/ligation reaction. Assembly of the genetic parts into the destination vector is strongly favored against religation into the source vector, since Type IIS restriction sites are lost in the assembled product [32]. In a second step, several transcription units in level 1 vectors can be released by cleavage with BbsI and assembled in a single step into a level 2 destination vector present in the same reaction. This second assembly step allows the construction of multigene clusters [29, 30]. The MoClo strategy with libraries of standardized parts has already been adopted for several organisms, including vascular plants [33], *Chlamydomonas reinhardtii* [35], *Phaeodactylum tricornutum* [36], *Saccharomyces cerevisiae* [37, 38], cyanobacteria [39], or proteobacteria [40]. MoClo is commonly applied also in the worldwide international Genetically Engineered Machine (iGEM) competition in which teams of supervised students present their applications in synthetic biology (competition.igem.org).

Here, we formed a supervised iGEM team of bachelor students at the TU Kaiserslautern (2021.igem.org/Team:TU_Kaiserslautern) and established a MoClo toolkit for the one-step assembly of predefined genetic parts that greatly facilitates the generation of constructs for systematic protein targeting or the production of recombinant proteins in *L. tarentolae*. The kit provides 34 genetic parts encoding various affinity tags, targeting signals as well as fluorescent and luminescent proteins. We tested 20 of these parts and exemplified the utility of the toolkit for the production and purification of the receptor binding domain (RBD) of the SARS-CoV-2 spike protein.

## RESULTS

### Generation of MoClo recipient vector and genetic parts

**Figure 1:**
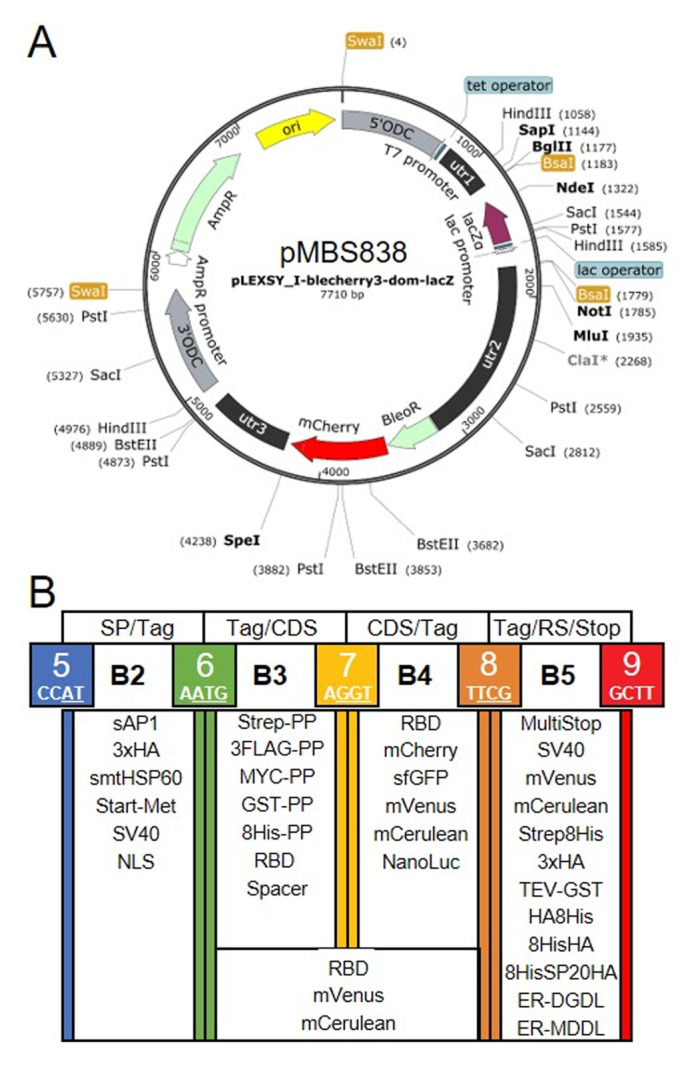
FIGURE 1: Genetic parts of the *L. tarentolae* MoClo kit. **(A)** Map of the destination vector for the *L. tarentolae* MoClo kit based on the pLEXSY_I-blecherry3 plasmid from Jena Bioscience. BsaI restriction sites were removed and the coding region for *lacZα* flanked by BsaI restriction sites was introduced to yield CCAT and GCTT fusion sites upon digestion with BsaI. **(B)** List of 34 Level 0 MoClo parts for positions B2 to B5 that are compatible with the MoClo syntax for plants and algae. The color code for fusion sites was adopted from the Chlamydomonas MoClo kit [35]. Nucleotides used in codons are underlined in white. SP, signal peptide; CDS, coding sequence; RS, retention signal; sAP1, secreted acid phosphatase 1; HA, human influenza hemagglutinin; Met, methionine; Strep, streptavidin; PP, PreScission protease cleavage site; GST, glutathione transferase; RBD, receptor binding domain of SARS-CoV-2; SV40, simian-virus 40; TEV, tobacco etch virus protease cleavage site; SP, serine-proline repeat; ER, endoplasmic reticulum.

Our goal was to establish a Modular Cloning system for the production of recombinant proteins in *L. tarentolae*. To this end, we first had to find a suitable expression vector. We chose the LEXSY expression system from Jena Bioscience, specifically the pLEXSY_I-blecherry3 plasmid. In this vector, target genes are inserted into an expression cassette with a T7 promoter that is under the control of a tetracycline (TET) operator, thereby featuring inducible, high-level expression (**Figure 1A**). The vector is introduced into an engineered *L. tarentolae* expression host that constitutively expresses T7 RNA polymerase and a TET repressor [23]. The expression cassette can integrate into the chromosomal ornithine decarboxylase (*odc*) locus via the 5'- and 3'-*ODC* homology regions (**Figure 1A**). The *blecherry* fusion gene allows selection on bleomycin and screening of the most productive clones based on mCherry fluorescence. We first domesticated the pLEXSY_I-blecherry3 plasmid by removing three internal BsaI restriction sites. Next, we introduced BsaI sites flanking the expression cassette and exchanged the stuffer sequences by the *lacZα* fragment to enable blue-white color selection in *E. coli*. Digestion of the resulting pLEXSY_I-blecherry3-dom-lacZ plasmid with BsaI results in removal of the *lacZα* fragment and creation of CCAT (5') and GCTT (3') overhangs (**Figure 1A and B**). These represent the flanking sites of positions B2 and B5, respectively, of the MoClo syntax established for plants and algae [34–36]. In this syntax, positions A1-3 are for promoter parts, B1 for the 5'UTR, B2 for parts encoding an N-terminal signal peptide and/or tag, B3/4 for coding sequences, B5 for parts encoding a C-terminal tag, B6 for the 3'UTR, and C1 for the terminator. Since the pLEXSY vector already contains promoter, 5'UTR, 3'UTR, and terminator, only positions B2 to B5 were relevant for our purpose. These positions allow for the one-step assembly of up to four predefined genetic parts (level 0) encoding target proteins, affinity tags, fluorescent proteins, a bioluminescent reporter, and signals for secretion or targeting to other subcellular compartments. Overall, we provide 34 genetic parts in our *L. tarentolae* MoClo kit, which are listed in **Figure 1B** and **Table 1**. Most of these parts were synthesized *de novo* with optimal *L. tarentolae* codon usage.

**Table 1.** List of level 0 parts for the *L. tarentolae* MoClo system.

**Plasmid**	**Part name**	**Pos**	**Function**	**Source**
pMBS-839	sAP1	B2	Protein secretion	PCR with pLEXSY_I-blecherry3 as template and primers SAP1-for/rev
pMBS-840	3xHA	B2	Protein detection	PCR with pCM0-100 as template [35] and primers HA-B2-for/rev
pMBS-843	smtHSP60*	B2	Protein targeting to mitochondrial matrix	Annealing of oligos MiMa-B2-for/rev (codon-optimized sequence coding for 19 N-terminal amino acids of mtHSP60 from *L. tarentolae*
pMBS-1125	Start-Met*	B2	Start methionine	Annealing of oligos Met-B2-for/rev (introduces MetAlaMet at N-terminus
pCM0-054	SV40	B2	Nuclear localization signal SV40	*Chlamydomonas* MoClo kit [35]
pMBS-844	NLS	B2	Nuclear localization signal nucleoplasmin	Annealing of oligos NLS-B2-for/rev (codon-optimized sequence
pMBS-845	Strep-PP*	B3	Protein purification and cleavage	IDT gBlocks (codon-optimized sequence coding for a Strep tag linked via RGSG to the LEVLFQ/G cleavage site for PreScission Protease
pMBS-846	3xFLAG-PP*	B3	Protein purification and cleavage	IDT gBlocks (semi codon-optimized sequence coding for three consecutive FLAG tags linked via RGS to the PreScission Protease cleavage site
pMBS-847	Myc-PP	B3	Protein purification and cleavage	IDT gBlocks (codon-optimized sequence coding for a Myc tag linked via RQS to the PreScission Protease cleavage site
pMBS-848	GST-PP*	B3	Protein purification and cleavage	IDT gBlocks (codon-optimized sequence coding for the 220 amino-acids GST with a C-terminal PreScission Protease cleavage site
pMBS-849	8xHis-PP*	B3	Protein purification and cleavage	IDT gBlocks (semi codon-optimized sequence coding for 8xHis linked via GS to the PreScission Protease cleavage site
pMBS-851	Spacer	B3	Bridges B3	Annealing of oligos Spacer-B3-for/rev (bridges B3 with sequences coding for MSGGGGG
pMBS-850	RBD	B3	Receptor binding domain of SARS-CoV-2	PCR amplification with pMBS857 as template and primers RBD-B3-for/rev
pMBS-857	RBD	B3/4	Receptor binding domain of SARS-CoV-2	IDT gBlocks (codon-optimized sequence coding for 223-amino-acids SARS-CoV-2 RBD
pMBS-858	mVenus*	B3/4	Fluorescent protein	PCR amplification with pCM0-066 as template [35] and primers mVe-for/mVeIn-up, and primers mVeIn-down/mVe-rev to remove intron from sequence and bring it to position B3/B4
pMBS-859	mCerulean*	B3/4	Fluorescent protein	PCR amplification with pCM0-046 as template [35] and primers mCe-for/mCeIn-up, and primers mCeIn-down/mCe-rev to remove intron from sequence and bring it to position B3/B4
pMBS-852	RBD	B4	Receptor binding domain of SARS-CoV-2	PCR amplification with pMBS857 as template and primers RBD-B4-for/rev
pMBS-855	mCherry*	B4	Fluorescent protein	IDT gBlocks (codon-optimized sequence coding for 236-amino-acids mCherry
pMBS-856	sfGFP	B4	Fluorescent protein	IDT gBlocks (codon-optimized sequence coding for 238-amino-acids superfolder GFP
pMBS-854	mVenus	B4	Fluorescent protein	PCR amplification with pMBS858 as template and primers mVe-B4-for/rev
pMBS-853	mCerulean	B4	Fluorescent protein	PCR amplification with pMBS859 as template and primers mCe-B4-for/rev
pMBS-872	NanoLuc	B4	Luminescence reporter	IDT gBlocks (codon-optimized sequence coding for 171-amino-acids NanoLuc Luciferase
pCM0-101	Multi-Stop*	B5	Adds 3 stops for each frame	*Chlamydomonas* MoClo kit [35]
pCM0-109	SV40-Stop*	B5	Nuclear localization signal SV40	*Chlamydomonas* MoClo kit [35]
pMBS-860	mVenus-Stop	B5	Fluorescent protein	PCR amplification with pMBS858 as template and primers mVe-B5-for/rev
pMBS-861	mCerulean-Stop	B5	Fluorescent protein	PCR amplification with pMBS859 as template and primers mCe-B5-for/rev
pCM0-99	Strep-8xHis-Stop	B5	Protein-purification	*Chlamydomonas* MoClo kit [35]
pCM0-100	3xHA-Stop	B5	Protein detection	*Chlamydomonas* MoClo kit [35]
pMBS-864	TEV-GST-Stop	B5	Protein-purification	IDT gBlocks (codon-optimized sequence coding for the 220 amino-acids GST with an N-terminal ENLYFQ/S TEV cleavage site
pMBS-723	1xHA-8xHis-Stop	B5	Protein detection and purification	Extended *Chlamydomonas* MoClo kit [54]
pMBS-871	8xHis-1xHA-Stop	B5	Protein detection and purification	Annealing of oligos 8HisHA-B5-for/rev
pMBS-1022	8xHis-SP20–3xHA	B5	Protein detection and purification	PCR amplification with pMBS659 as template and primers 8HisSPHA-for/rev
pMBS-869	ER-DGDLStop*	B5	ER retention signal	Annealing of oligos DGDL-B5-for/rev
pMBS-870	ER-MDDLStop*	B5	ER retention signal	Annealing of oligos MDDL-B5-for/rev

* These modules remain to be tested in *L. tarentolae*.

Since the latter is very similar to that of *Chlamydomonas reinhardtii* (with a bias for C in the third position in *Chlamydomonas*), we could directly use six parts for short tags from the *Chlamydomonas* MoClo kit [35, 54], demonstrating the interchangeability of standardized parts with the MoClo system. Sequences coding for mCerulean and mVenus were also taken from the *Chlamydomonas* MoClo kit, but introns had to be removed by PCR. Successful assembly of level 0 parts into the level 1 destination vector pLEXSY_I-blecherry3_dom_lacZ (pMBS838) can be monitored by blue-white color selection. The expression cassette from the resulting level 1 plasmids isolated from positive (white) colonies can be excised by SwaI (**Figure 1A**) and directly transfected into the *L. tarentolae* host.

### Successful production of secreted recombinant RBD

**Figure 2:**
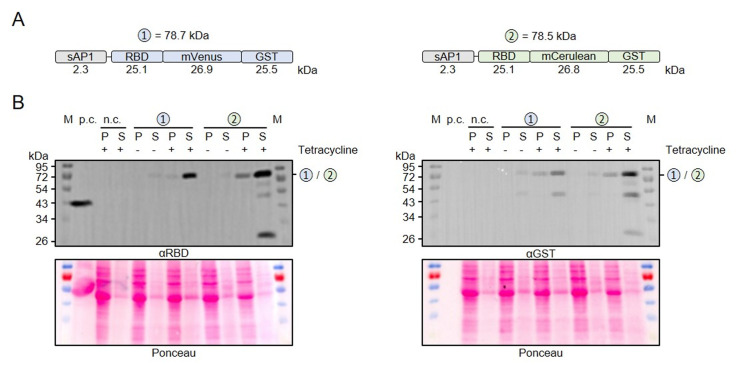
FIGURE 2: Production and secretion of recombinant RBD fusion proteins. **(A)** Schematic overview and expected mass of the secreted RBD fusion proteins. **(B)** Comparative Western blot analysis with antibodies against the RBD domain (left) and the GST domain (right) of RBD fusion proteins in the cell-containing pellet fraction (P) and supernatant fraction (S) following tetracycline induction of the according *L. tarentolae* liquid cultures. Recombinant RBD served as positive control (p.c.) and an induced culture without plasmid as negative control (n.c.). The calculated masses from panel A are indicated.

To test our MoClo system, we chose the receptor-binding domain (RBD) from the SARS-CoV-2 spike protein as a target. RBD contains two *N*-glycans [41] whose formation requires the protein to pass through the secretory pathway. We therefore assembled a level 1 module to produce RBD with an N-terminal signal peptide from *Leishmania donovani* secreted acid phosphatase (sAP1) [42] and C-terminal fusions between mVenus or mCerulean and glutathione transferase from *Schistosoma japonicum* (mVenus-GST or mCerulean-GST, respectively) (**Figure 2A**). Both recombinant proteins were successfully produced in *L. tarentolae* liquid cultures following the addition of tetracycline (**Figure 2B**). Western blot analyses with antibodies against the RBD and the GST domain both revealed a weak signal around 80 kDa in the cell-containing pellet fraction and a strong signal in the supernatant fraction, indicating the functionality of the N-terminal signal peptide and successful secretion of both fusion proteins into the medium. Additional weaker bands around 27, 52, and 65 kDa in both blots revealed a limited proteolysis between the RBD, the fluorescent protein, and the two GST domains in accordance with folded individual protein domains. In summary, we confirmed the successful production and secretion of two tagged versions of RBD in *L. tarentolae* using our MoClo kit.

### Limited proteolysis and artificial *O*-glycosylation of secreted recombinant RBD

**Figure 3:**
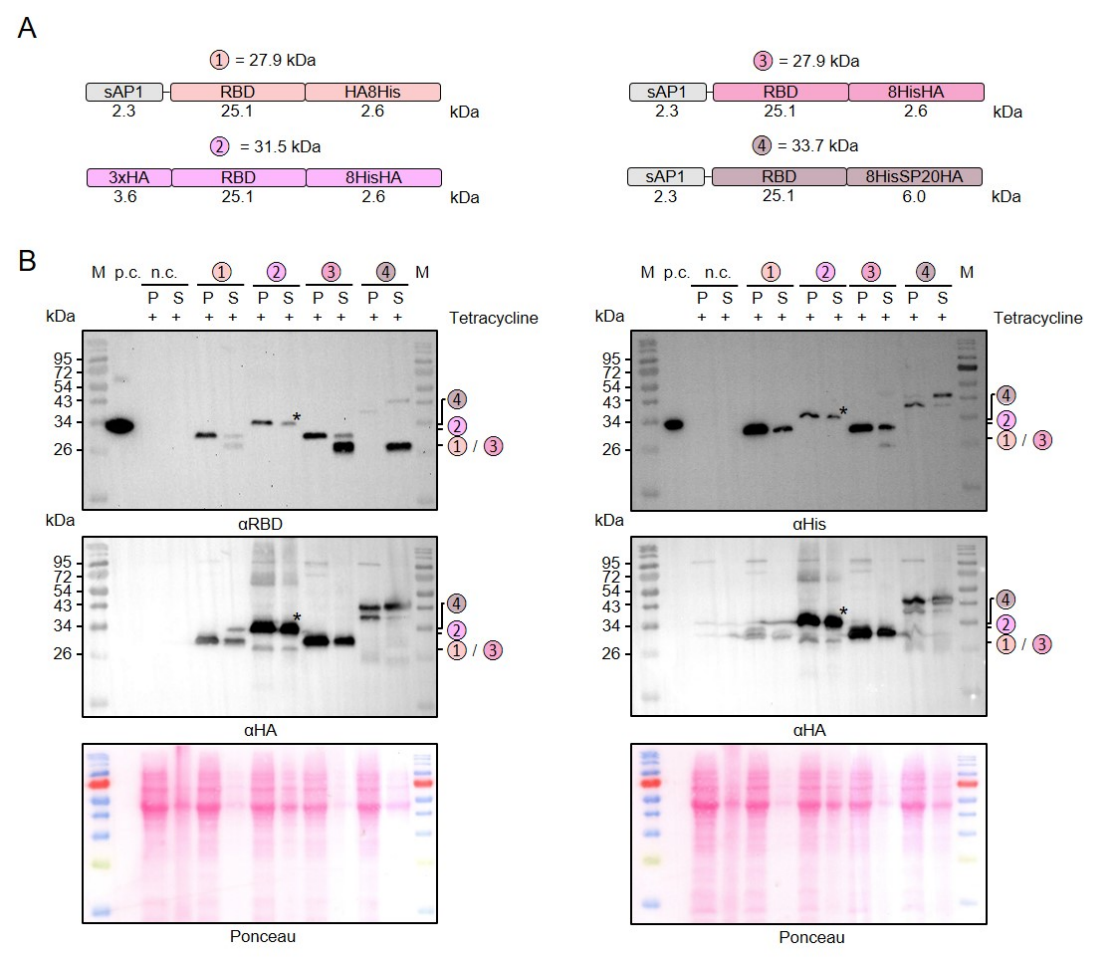
FIGURE 3: Limited proteolysis and glycosylation of secreted C-terminally tagged recombinant RBD. **(A)** Schematic overview and expected mass of the secreted RBD variants with swapped C-terminal peptide tags. Variant 2 with an N-terminal 3xHA tag instead of the N-terminal signal peptide served as a cytosolic control. Variant 4 also contains an SP20 sequence for extensive *O*-glycosylation. **(B)** Comparative Western blot analysis with antibodies against the RBD domain (left) or the 8xHis tag (right) in the cell-containing pellet fraction (P) and supernatant fraction (S) following tetracycline induction of the according *L. tarentolae* liquid cultures. An induced culture without plasmid served as negative control (n.c.) and recombinant His-tagged RBD with an expected size of 35 kDa as positive control (p.c.). The asterisk labels a band that was caused by a contamination of the supernatant fraction in the experiment shown. Both membranes were stripped and redecorated with an antibody against the HA tag. The calculated masses from panel A are indicated.

Next, we used our MoClo kit to analyze the effect of different tags on the stability of secreted recombinant RBD. Comparisons between thirteen different heterologous versions of RBD revealed 1) that C-terminal peptide tagging tended to result in lower yields of soluble protein than C-terminal GST tagging and 2) that C-terminal peptide tags, including 8xHis, Strep, or HA tags, were usually partially or even fully degraded in the supernatant fraction (Supplementary Figures S1 and S2). A partial degradation of the C-terminal HA-8xHis tag was also observed for a secreted heterologous luciferase (NanoLuc), suggesting a general peptidase activity outside the cell independent of the tagged protein (Supplementary Figure S2). We then analyzed a potential limited proteolysis for a cytosolic control and for proteins with swapped positions of the peptide tags (**Figure 3A**). While swapping the position of the HA and the 8xHis tag increased the yield of secreted RBD, limited proteolysis still occurred when the HA tag was at the C-terminus (**Figure 3B**). In contrast to secreted RBD, C-terminally HA-8xHis-tagged cytosolic RBD was not processed. Addition of PMSF or of cOmplete™ Protease Inhibitor Cocktail to the medium or the Laemmli buffer had no effect on the limited proteolysis, which might be explained by a continuous replacement of inactivated peptidases during the induction period or by an insensitivity of the responsible peptidase to these inhibitors (data not shown). The C-terminal peptide tag was also predominantly degraded for secreted RBD with a tripartite tag consisting of an 8xHis tag, a synthetic *O*-glycosylation module with twenty Ser-Pro repeats (SP20) [43], and an HA tag. However, a fraction of the secreted 8xHis-SP20-HA-tagged RBD was successfully glycosylated as indicated by apparent mass shifts of approximately 6 and 10 kDa (**Figure 3B**). While the less glycosylated protein with an apparent mass shift of 6 kDa was more prominent in the cell pellet fraction, the glycosylated protein with a mass shift of 10 kDa predominated in the supernatant fraction, suggesting a stepwise glycosylation in the Golgi apparatus.

In summary, we successfully employed our versatile MoClo kit to analyze the effect of different tags on the stability of secreted RBD in *L. tarentolae*. Our data point towards an unspecific extracellular peptidase activity in *L. tarentolae* cultures that caused limited proteolysis, regardless of whether there was an 8xHis, Strep, or HA tag at the C-terminus of the secreted recombinant protein. C-terminal GST-tagging correlated with an increased yield of secreted recombinant RBD and fusion to a synthetic SP20 peptide resulted in an apparent mass shift of 10 kDa in accordance with a successful *O*-glycosylation.

### Relevance of the induction period for the production of secreted recombinant RBD

**Figure 4:**
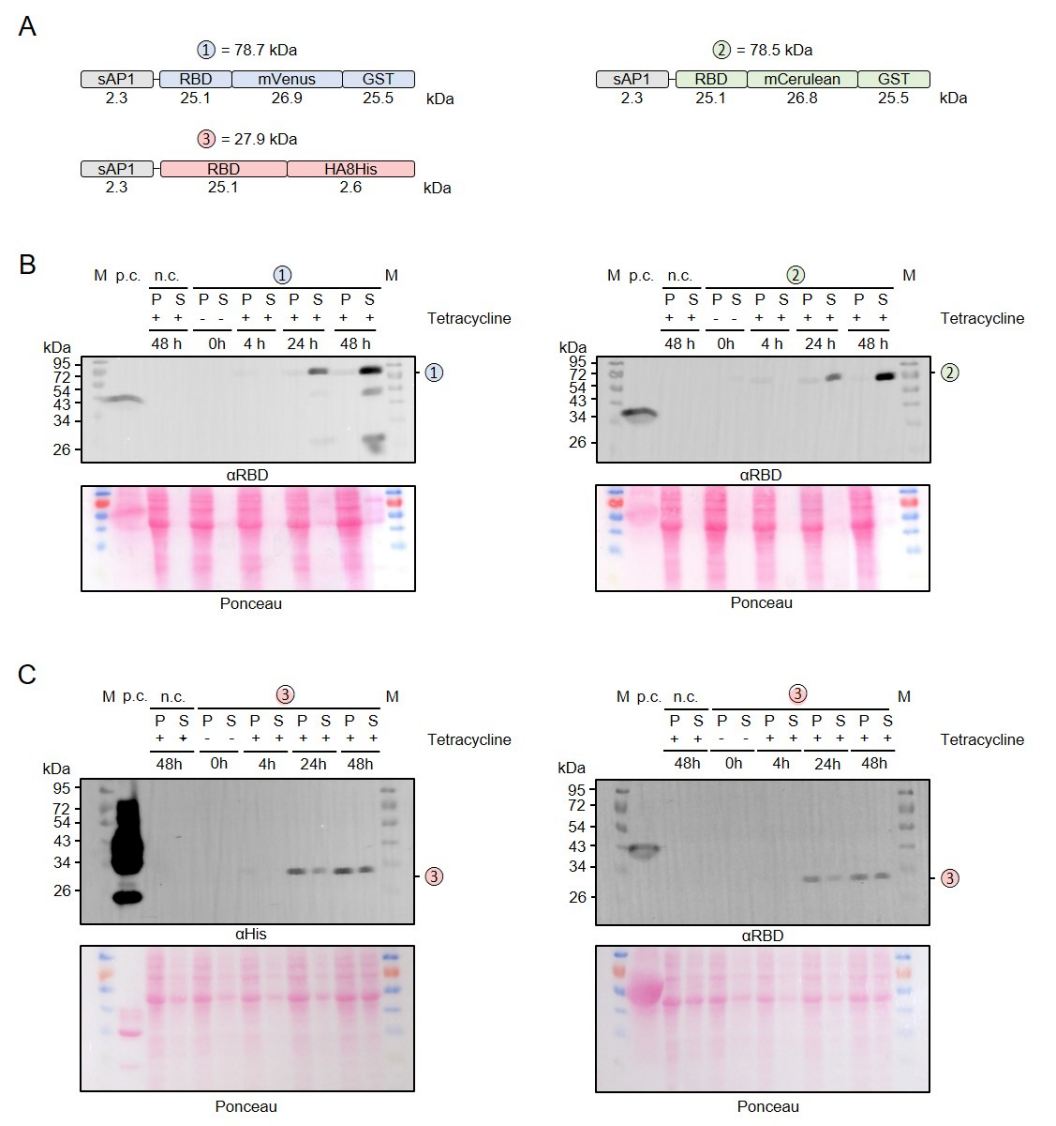
FIGURE 4: Time course measurement for the production and secretion of recombinant RBD. **(A)** Schematic overview and expected mass of the secreted RBD fusion proteins. **(B)** Western blot analysis with an antibody against the RBD domain of RBD fusion variants 1 and 2 in the cell-containing pellet fraction (P) and supernatant fraction (S) following tetracycline induction of the according *L. tarentolae* liquid cultures for the indicated time periods. An induced culture without plasmid served as negative control (n.c.) and recombinant RBD as positive control (p.c.). The calculated masses from panel A are indicated. **(C)** Western blot analysis with antibodies against the C-terminal His tag (left) or the RBD domain (right) of fusion variant 3.

To address a possible effect of the induction period on the limited proteolysis and yield of secreted recombinant RBD, we performed time course measurements for RBD that was fused with either mVenus-GST, mCerulean-GST or an HA-8xHis tag (**Figure 4A**). No recombinant protein was detected in uninduced cultures without tetracycline and the yields of all secreted RBD variants increased significantly between 24 and 48 h post induction (**Figure 4B, C**). While only very little intracellular GST-tagged protein was detectable (**Figure 4B**), the amount of HA-8xHis-tagged RBD in the cell pellet fraction was rather high at 24 and 48 h post induction (**Figure 4C**). Limited proteolysis of the mVenus-GST fusion protein reconfirmed its domain architecture and was already detectable at 24 h post induction (**Figure 4B**). In contrast to the mVenus-GST fusion protein and the results from **Figure 2B**, limited proteolysis was absent for the mCerulean-GST fusion protein. Thus, limited proteolysis appeared to rather depend on the chosen *L. tarentolae* clone and could not be prevented by shorter induction periods. In summary, a prolonged induction for 48 h increases the yield of secreted RBD fusion proteins.

### Relevance of antibiotics for the production of secreted recombinant RBD

**Figure 5:**
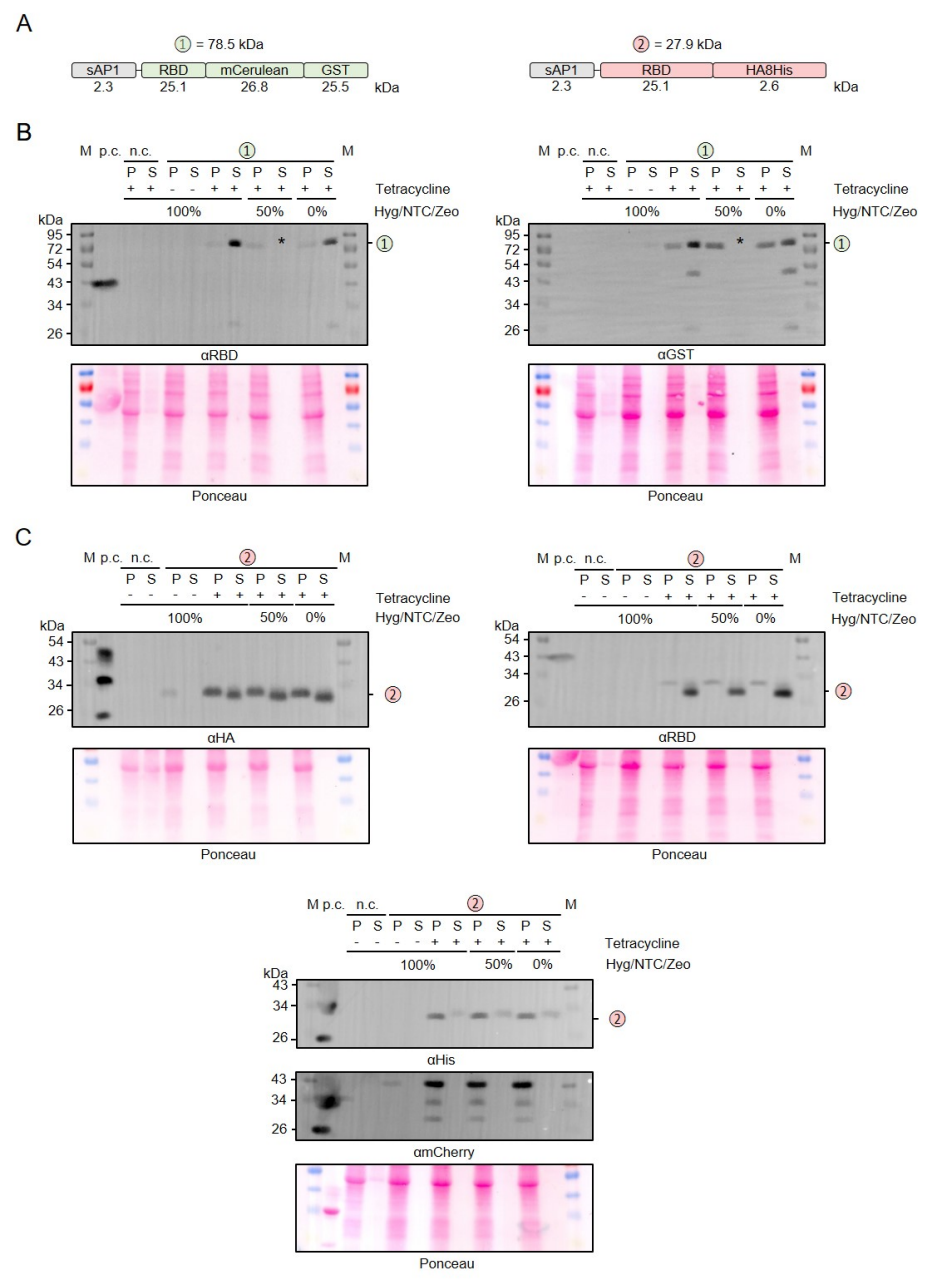
FIGURE 5: Relevance of antibiotics for the production and secretion of recombinant RBD. **(A)** Schematic overview and expected mass of the secreted RBD fusion proteins. **(B)** Western blot analysis with antibodies against the RBD domain (left) and the GST domain (right) of RBD fusion variant 1 in the cell-containing pellet fraction (P) and supernatant fraction (S) following tetracycline induction of the according *L. tarentolae* liquid cultures containing the indicated amounts of antibiotics. An induced culture without plasmid served as negative control (n.c.) and recombinant RBD as positive control (p.c.). The asterisk labels the TCA precipitate of the 50% supernatant fraction which was lost in the experiment shown. The calculated mass from panel A is indicated. **(C)** Western blot analysis with antibodies against the HA tag (left), the RBD domain (right), and the His tag (bottom) of RBD fusion variant 2.

The adapted LEXSY system requires the selection with three different antibiotics to maintain stable *L. tarentolae* strains. Since this has a large impact on the production cost for recombinant proteins in *L. tarentolae* and the total amount of antibiotics to be used, we tested whether the antibiotics affect the protein production following tetracycline induction (**Figure 5**). We compared the production and secretion of RBD that was fused to either mCerulean-GST or an HA-8xHis tag (**Figure 5A**) in the presence or absence of hygromycin (Hyg), nourseothricin (NTC), and zeocin (ZEO) (**Figure 5B, C**). Reducing the antibiotic concentrations to 50% or even omitting all antibiotics during the 48-h induction phase had no effect on the overall yield of secreted protein. Furthermore, omitting the antibiotics had no effect on the limited proteolysis between the folded domains (**Figure 5B**), the detected degradation of the C-terminal 8xHis tag, or the cell integrity as revealed by the absence of mCherry in the supernatant fractions (**Figure 5C**). In summary, all three antibiotics can be omitted during the induction phase without any disadvantage on the production of secreted recombinant RBD.

### Purification of secreted recombinant RBD

**Figure 6:**
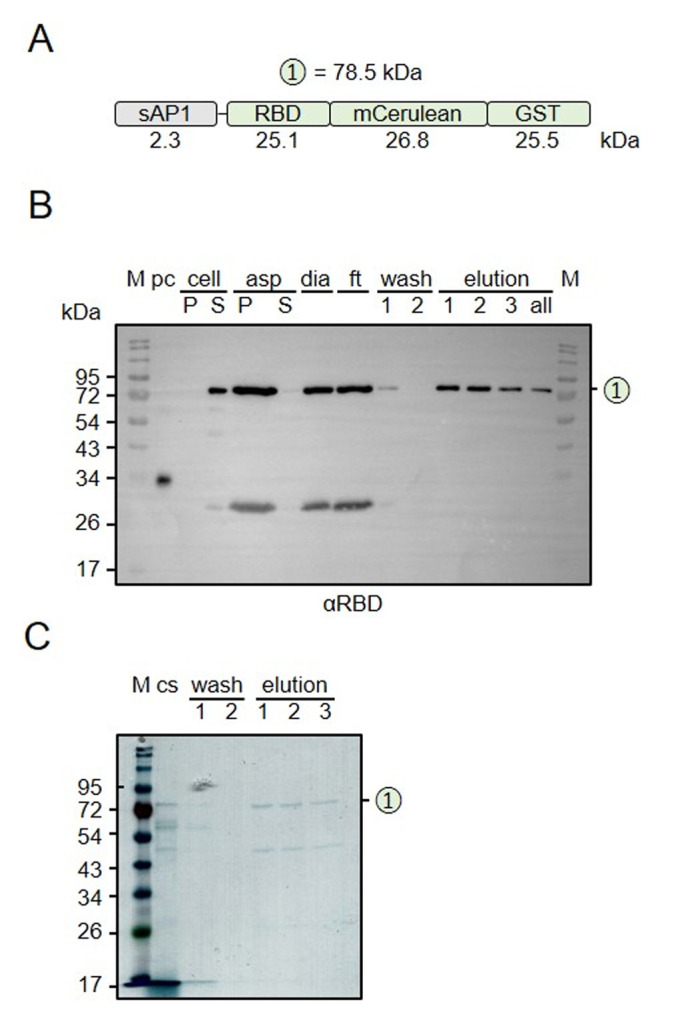
FIGURE 6: Purification of secreted GST-tagged RBD. **(A)** Schematic overview and expected mass of the secreted RBD protein. **(B)** Western blot analysis with an antibody against the RBD domain. Recombinant RBD served as a positive control (p.c.). Cell, cell culture; P, pellet (10^7^ cells); S, supernatant (0.2%); asp, ammonium sulfate precipitation pellet (0.13%) and supernatant (0.13%); dia, dialysis (0.07%); ft, flow through (0.07%); wash, wash steps (0.08%); elution, eluate fractions (0.8%); all, pooled eluate; **(C)** SDS-PAGE analysis of the cell culture supernatant (cs), the wash, and eluate fractions of the affinity chromatography from panel B. The gel was stained with Coomassie blue.

To determine whether secreted recombinant RBD can be purified from *L. tarentolae* cultures, we carried out pilot experiments for mCerulean-GST-tagged RBD and established a first purification protocol (**Figure 6**). The supernatant of an induced culture was precipitated with ammonium sulfate, resuspended, dialyzed, and purified by affinity chromatography via the GST tag (**Figure 6B**). While several purification parameters remain to be optimized, for example, to avoid that most of the GST-tagged protein ends up in the flow-through fraction, Western blot and SDS-PAGE and Coomassie staining analyses revealed a successful purification of the whole fusion protein with the expected mass around 78 kDa (**Figure 6B, C**). A 50 kDa protein, presumably corresponding to GST-tagged mCerulean, co-eluted from the glutathione sepharose column and was detected by SDS-PAGE/Coomassie staining but not by Western blot analysis. In summary, RBD can be purified from the supernatant of *L. tarentolae* liquid cultures using a correctly folded GST domain for affinity chromatography.

### Systematic protein targeting

We also tested the suitability of our MoClo toolkit for systematic intracellular protein targeting in *L. tarentolae* (**Table 1**). As a proof of principle, we analyzed the localization of a heterologous superfolder variant of green fluorescent protein (sfGFP) that was fused at the N-terminus to either the monopartite nuclear localization signal (NLS) of simian virus 40 (which is also used for the heterologous T7 RNA polymerase [23] and served as a positive control) or the bipartite NLS of nucleoplasmin from *Xenopus* [44] (Supplementary Figure S3). The GFP fluorescence patterns were compared to the fluorescence of plasmid-encoded cytosolic mCherry and the DAPI fluorescence for nuclear and kinetoplast DNA. Parasites containing the empty vector pLEXSY_I-blecherry3_dom_lacZ served as a negative control. Signals for mCherry and DAPI but not for GFP were detected in the negative control, thus excluding potential background signals from the mitochondrial autofluorescence [45]. In contrast to the negative control, GFP signals were detected for both NLS-tagged sfGFP versions. The GFP fluorescence co-localized with the nuclear but not the kinetoplast DAPI fluorescence in accordance with a successful nuclear import. In summary, while several of our localization modules remain to be tested (**Table 1**), successful targeting of sfGFP to the nucleus confirms the general suitability of our MoClo toolkit for systematic intracellular protein targeting in *L. tarentolae*.

## DISCUSSION

We report here on the establishment of a MoClo toolkit for the production of recombinant proteins in *L. tarentolae*. The system is based on the LEXSY expression system using a vector that provides a tetracycline-inducible promoter and is stably integrated into the genome. Our engineered destination vector (pMBS838) allows the one-step assembly of genetic parts in up to four positions with blue/white color selection in *E. coli* for successful part assembly. We provide 34 domesticated genetic parts in level 0 vectors. Our system allows for 1) the use of various affinity tags (HA, FLAG, Myc, 8xHis, GST, Strep) for N- and C-terminal fusions, 2) alternative purification strategies from cells (intracellular localization) or culture medium (secretion), 3) the use of various fluorescence markers (sfGFP, mCerulean, mVenus, mCherry) and a luminescent reporter (NanoLuc) for N- and C-terminal fusions, and 4) the targeting to various intracellular compartments (cytosol, mitochondrial matrix, ER, nucleus). Our toolkit follows the MoClo standard established for plants and algae [34–36], which allows an inter species exchange of parts. Here, for example, we employed the Strep, 8xHis, and HA tags developed for *C. reinhardtii* in *L. tarentolae* (**Figures 3-5**, Supplemental Figures 1 and 2). Moreover, we have verified the utility of several new parts and could demonstrate the successful production and secretion of the RBD of the SARS CoV-2 spike protein in various fusions with fluorescent proteins and affinity tags. The RBD is required for the binding of SARS CoV-2 to the angiotensin-converting enzyme 2 to initialize the entry of the virus into the human host cell [46, 47]. Recombinant RBD, which has been used for structure-function analysis, vaccination strategies, as well as diagnostic and therapeutic applications [47–50], has been produced in alternative cell systems including human embryonic kidney cells [47, 50], insect cells [48], *Nicotiana benthamiana* [51], *Pichia pastoris* [52], and *C. reinhardtii* [53, 54]. Our data show that folded RBD can be also produced and purified from *L. tarentolae* liquid cultures. The fusion of RBD to the SP20 *O*-glycosylation module resulted in an altered electrophoretic mobility in accordance with a successful glycosylation but did not increase the yield of secreted protein in contrast to reports in land plants and *C. reinhardtii* [43, 54, 55]. While the fused SP20 module confirmed the functionality of the glycosylation machinery in *L. tarentolae*, other recombinant RBD fusion variants were detected at the predicted molecular mass. Thus, although we cannot exclude some degree of glycosylation of our recombinant RBD, more pronounced *N*- and *O*-glycosylation of RBD, as reported previously [47, 49, 56, 57], appears to be absent in *L. tarentolae*. We also showed that a prolonged 48-h induction period is optimal for an increased yield of secreted RBD and that the antibiotics can be omitted during the induction phase without affecting protein production. The latter observation could also be relevant for the production of other recombinant proteins, further improving the sustainability and cost balance of the LEXSY expression system. Another potentially transferable result of our study is that *L. tarentolae* possesses high peptidase and protease activity leading to the truncation of C-terminal peptide tags and/or cleavage between the folded domains of secreted proteins. We therefore suggest to either use a C-terminal GST tag, whose functionality we have been able to demonstrate by affinity chromatography for secreted RBD, or to move short affinity tags to more internal positions. In summary, we established the MoClo toolkit and exemplified its application for the modular production of recombinant proteins in *L. tarentolae* using the LEXSY expression system.

Future applications of our MoClo toolkit could be also extended to biochemical analyses in *L. tarentolae* or other (pathogenic) *Leishmania* spp, provided that these strains contain a constitutively expressed T7 RNA polymerase and a TET repressor. For example, genetically encoded calcium-, pH- or metabolite sensors [58–63] could be systematically targeted to different subcellular compartments, similar to experiments in yeast [64], *C. reinhardtii* [65], *A. thaliana* [66], *P. falciparum* [67] or mammalian cells [68]. Systematic Golden Gate cloning of *Leishmania* open reading frames into our MoClo vector could also be applied for subcellular localization studies using our N- or C-terminal (fluorescent protein) tags. While the LeishGEdit technology by Gluenz and colleagues [20] is probably the method of choice for comprehensive whole genome analyses in *Leishmania*, our MoClo system allows the rapid comparison of a variety of tags and could therefore complement the technology. Results from such systematic localization studies in *Leishmania* could not only add to the genome-wide subcellular protein map for *Trypanosoma brucei* and the TrypTag resource [69, 70] but also provide insights regarding similarities and differences between trypanosomatid lineages.

## MATERIALS AND METHODS

### Modular Cloning kit

*Domestication of pLEXSY_I-blecherry3 plasmid*. Five primer pairs were designed to amplify five fragments of the 8.2 kb pLEXSY_I-blecherry3 plasmid (Jena Bioscience). All primers introduced flanking BsaI restriction sites, giving rise to unique 4-nt overhangs upon digestion with BsaI (Supplementary Table 2). Primer pairs plex1-for/rev, plex2-for/rev, and plex3-for/rev also introduced point mutations to destroy internal BsaI sites. The five fragments were assembled by combined action of BsaI and T4-DNA ligase using the following Golden Gate reaction cycle: 13 x [37°C for 2 min, 16°C for 5 min], 50°C for 5 min, 80°C for 10 min. The fragment amplified with plex2-for/plex400-rev always missed some G/C-rich and repetitive sequences. This problem was fixed by exchanging the faulty region in the domesticated plasmid by a 1970-bp ClaI/SpeI fragment from the original pLEXSY_I-blecherry3 plasmid. To equip the domesticated vector with *lacZα* for blue-white color selection, a 623-bp *lacZα* fragment was amplified by PCR from plasmid pICH47742 [29]. The primers used introduced flanking BglII and NotI restriction sites and internal BsaI recognition sites, giving rise to CCAT and GCTT overhangs upon BsaI digestion. The resulting PCR product was digested with BglII and NotI and ligated into the BglII/NotI-digested domesticated vector, yielding the 7710-bp destination vector pLEXSY_I-blecherry3_dom_lacZ (pMBS838). The correct sequence was verified by Sanger sequencing (SeqLab, Göttingen).

*Level 0 parts*. Genetic parts for level 0 were synthesized with optimized *L. tarentolae* codon usage and flanking BbsI restriction sites by Integrated DNA Technologies (gBlocks), amplified by PCR using the primers listed in Supplementary Table 1 that introduced flanking BbsI restriction sites, or generated by the annealing of oligos that produced overhangs compatible with the respective cloning positions (Supplementary Table 1). Using the Golden Gate reaction cycle with BbsI and T4-DNA ligase, all parts were ligated into vectors pAGM1276 (B2), pICH41258 (B3), pAGM1299 (B4), pAGM1301 (B5), and pAGM1287 (B3-4) from the Weber collection [29]. Ligation products were transformed into *E. coli* TOP10 cells via heat shock and selected on LB plates containing spectinomycin (100 µg/mL), IPTG (0.5 mM), and X-Gal (40 µg/mL). Correct cloning was verified by restriction digestion and Sanger sequencing (SeqLab, Göttingen). All 34 level 0 parts and the MoClo destination vector pMBS838 can be ordered from Addgene (ID 217994 - 218022; 218199 - 218204).

*Level 1 modules*. Level 0 parts covering the B2-B5 cloning positions were assembled into the pMBS838 destination vector via BsaI and T4-DNA ligase using the Golden Gate reaction cycle. Transformed *E. coli* TOP10 cells were plated on LB plates with 100 µg/mL Ampicillin, IPTG and X-Gal. Correct cloning was verified by restriction digestion and Sanger sequencing (SeqLab, Göttingen). All PCR reactions were carried out with Q5® High-Fidelity DNA Polymerase (NEB). Purification of PCR products and plasmids was done with the NucleoSpin® Gel and PCR Clean-up kit and the NucleoSpin® Plasmid Easy Pure kit (Macherey-Nagel). DNA concentrations were determined using a NanoDrop spectrophotometer.

### Cultivation, transfection, and selection of *L. tarentolae*

*L. tarentolae* strain T7-TR promastigotes (Jena Bioscience) were cultured at 27°C in ventilated tissue culture flasks in an upright position on a Rotamax 120 shaker at 50 rpm in 10 mL brain heart infusion (BHI) medium according to standard protocols [6, 9, 18]. Unless otherwise stated, liquid cultures were grown in the presence of 5 µg/mL hemin, 100 µg/mL hygromycin, 100 µg/mL nourseothricin, 50 U/mL penicillin, and 50 µg/mL streptomycin (Jena Bioscience). Each transfection was carried out with 10^7^ parasites in mid-logarithmic phase that were washed with 1.0 mL transfection buffer (21 mM HEPES, 137 mM NaCl, 5 mM KCl, 0.7 mM NaH_2_PO_4_, 6 mM glucose, pH 7.4) and resuspended in 100-150 µL transfection buffer. Level 1 vectors were digested with SwaI to excise the expression cassette. The DNA (5-10 µg in 50 µL 5 mM Tris buffer pH 8.0) was incubated for 5 min at 95°C, slowly cooled, and mixed with the 100-150 µL cell suspension in a electroporation cuvette. Electroporation was carried out in a Lonza Nucleofector IIb using program X-001. Parasites were subsequently transferred to 1.0 mL hemin-containing BHI medium without antibiotics and incubated either overnight or until the suspension became turbid. Following a centrifugation at 1,000×*g* for 5 min at 27°C, cells were resuspended in 150 µL of the supernatant and plated on BHI agar plates containing 0.8% (w/v) agar, 10% (v/v) fetal bovine serum, 0.08% (w/v) folic acid, 20 µg/mL hemin, 100 µg/mL zeocin, and the same concentrations of hygromycin and nourseothricin as stated above. After selection, a nitrocellulose membrane was used to transfer colonies to agar plates containing 100 µg/mL tetracycline. Red colonies with a high mCherry content were identified after 24-48 h and transferred for subsequent expression studies to BHI liquid medium containing the indicated concentrations of hemin, hygromycin, nourseothricin, and zeocin.

### Expression studies and western blot analysis

*L. tarentolae* liquid cultures were grown for up to 48 h after the induction with 10 µg/mL tetracycline. To analyze the intracellular protein content, 10^8^ parasites were harvested by centrifugation at 3,000×*g* for 5 min at 27°C. The parasite pellet fraction was resuspended in 100 µL Laemmli buffer and boiled for 10 min at 97°C. Secreted proteins were analyzed from 10 mL cell culture supernatant following a centrifugation at 3,000×*g* for 10 min at 27°C. Proteins from the supernatant fraction were precipitated with 2.5 mL ice-cold 50% trichloroacetic acid for 30 min on ice before centrifugation at 15,000×*g* for 15 min at 4°C. The protein precipitate was washed three times with 1 mL ice-cold 80% acetone and was subsequently resuspended and boiled for 10 min at 97°C in 100 µL Laemmli buffer. Ten µL of each of the pellet and supernatant Laemmli samples were loaded on 10 or 15% SDS polyacrylamide gels and were separated by SDS-PAGE [71]. Proteins were transferred to methanol-activated polyvinylidene fluoride membranes by wet Western blotting and were subsequently stained with Ponceau S. Primary mouse antibodies were used for the immunodecoration of the HA-tag (Sigma Aldrich), the His-tag (Dianova), and the RBD (R&D systems and Thermofisher), whereas primary rabbit and goat antibodies were used for the immunodecoration of mCherry [35] and GST (Sigma-Aldrich), respectively. Horseradish-peroxidase-coupled secondary antibodies for enhanced chemiluminescence detection were from Santa Cruz Biotechnology.

### Protein purification

A 0.5 L culture of strain T7-TR with plasmid pLEXSY_I-blecherry3_dom_lacZ encoding secreted mCerulean-GST-tagged RBD was induced with 10 µg/mL tetracycline for 48 h and harvested by centrifugation at 4,000×*g* for 30 min at 4°C. The supernatant protein was slowly precipitated under constant stirring at 4°C by the stepwise addition of ammonium sulfate to a final concentration of 576 g/L. The dispersion was centrifuged at 4,000×*g* for 30 min at 4°C and the resulting precipitate was resuspended in 1.5 volumes (ca. 4 mL) equilibration buffer containing 150 mM NaCl, 50 mM Tris, HCl, pH 8.0 at 4°C. The sample was dialyzed in a 28 µm cellulose tubing with a Mw cut-off of 6,000 - 8,000 (Carl Roth) against 2×5 L equilibration buffer overnight at 4°C. One mL Pierce™ glutathione agarose (Thermo Fisher) was equilibrated with 10 mL equilibration buffer, centrifuged at 700×*g* for 2 min at 4°C, and subsequently incubated with the dialyzed protein sample on a rotator overnight at 4°C. The agarose was washed twice with 10 mL equilibration buffer before the stepwise elution with three times 1 mL elution buffer containing 10 mM GSH, 150 mM NaCl, 50 mM Tris, HCl, pH 8.0 at 4°C. Samples of each purification step were supplemented with Laemmli buffer, boiled for 10 min at 97°C, and analyzed by SDS-PAGE and Western blot analysis.

### Fluorescence microscopy

Parasites transfected with empty vector pLEXSY_I-blecherry3_dom_lacZ or L1 constructs containing either NLS-encoding sequence pMBS844 or pCM0-054 were cultured in mid-logarithmic phase and 5×10^6^ parasites were harvested by centrifugation at 3,000×g for 5 min at 27°C. Parasites were washed three times with 1 mL phosphate buffered saline (PBS) and incubated with 4% (w/v) formaldehyde in PBS for 15 min at room temperature. Fixed parasites were washed twice with PBS and subsequently mounted on a slide with one drop of ROTI®Mount FluorCare DAPI. The next day, slides were analyzed using a Nikon CSU-X1 spinning disc confocal microscope, an 63x oil objective, and the software NIS-elements. Three different laser excitation wavelengths of 405, 488, and 561 nm were used for the detection of DAPI, sfGFP, and mCherry respectively. Images were analyzed and processed using the software ImageJ [72].

## SUPPLEMENTAL MATERIAL

Click here for supplemental data file.

All supplemental data for this article are available online at www.microbialcell.com/researcharticles/2024a-hieronimus-microbial-cell/.

## Abbreviations:

MoClo: – modular cloning,
iGEM: – international genetically engineered machine,
RBD: – receptor binding domain,
UTR: – untranslated region,
TET: – tetracycline,
ODC: – ornithine decarboxylase,
sAP1: – secreted acid phosphatase,
Hyg: – hygromycin,
NTC: – nourseothricin,
ZEO: – zeocin,
sfGFP: – superfolder green fluorescent protein,
NLS: – nuclear localization signal,
BHI: – brain heart infusion,
PBS: – phosphate buffered saline.
