# Supplementary material for: A Modular Cloning Toolkit for the production of recombinant proteins in Leishmania tarentolae
Source: Microb Cell. 2024 Apr 30;11:128–42. doi: 10.15698/mic2024.04.821 (PMC11121976; doi:10.15698/mic2024.04.821)
Supplement: Supplementary file 1 [file mic-11-128-s01.pdf]

Supplementary information

A Modular Cloning Toolkit for the production of recombinant proteins in *Leishmania tarentolae*

Katrin Hieronimus[1,2,‡], Tabea Donauer[1,2,‡], Jonas Klein[1,‡], Bastian Hinkel[1,‡], Julia Vanessa Spänle[1,‡], Anna Probst[1,‡], Justus Niemeyer[1,‡], Salina Kibrom[1], Anna Maria Kiefer[1], Luzia Schneider[2], Britta Husemann[2], Eileen Bischoff[2], Sophie Möhring,[2] Nicolas Bayer[1], Dorothée Klein[1], Adrian Engels[1], Benjamin Gustav Ziehmer[1], Julian Stieß[3], Pavlo Moroka[1], Michael Schroda[1,]*, and Marcel Deponte[2,] *

[1]Faculty of Biology, Molecular Biotechnology & Systems Biology, RPTU Kaiserslautern, D-67663 Kaiserslautern, Germany

[2]Faculty of Chemistry, Comparative Biochemistry, RPTU Kaiserslautern, D-67663 Kaiserslautern, Germany

[3]Faculty of Computer Science, RPTU Kaiserslautern, D-67663 Kaiserslautern, Germany

[‡] Authors contributed equally to this work

*Correspondence to: Michael Schroda

Faculty of Biology, Molecular Biotechnology & Systems Biology, RPTU Kaiserslautern, D-67663 Kaiserslautern, Germany

Email: schroda@bio.uni-kl.de

Phone: +49 631 205 2697

Fax: +49 631 205 3799

*Correspondence to: Marcel Deponte

Faculty of Chemistry, Comparative Biochemistry, RPTU Kaiserslautern, D-67663 Kaiserslautern, Germany

Email: deponte@chemie.uni-kl.de

Phone: +49 631 205 3421

Fax: +49 631 205 3419

Supplementary Figure S1

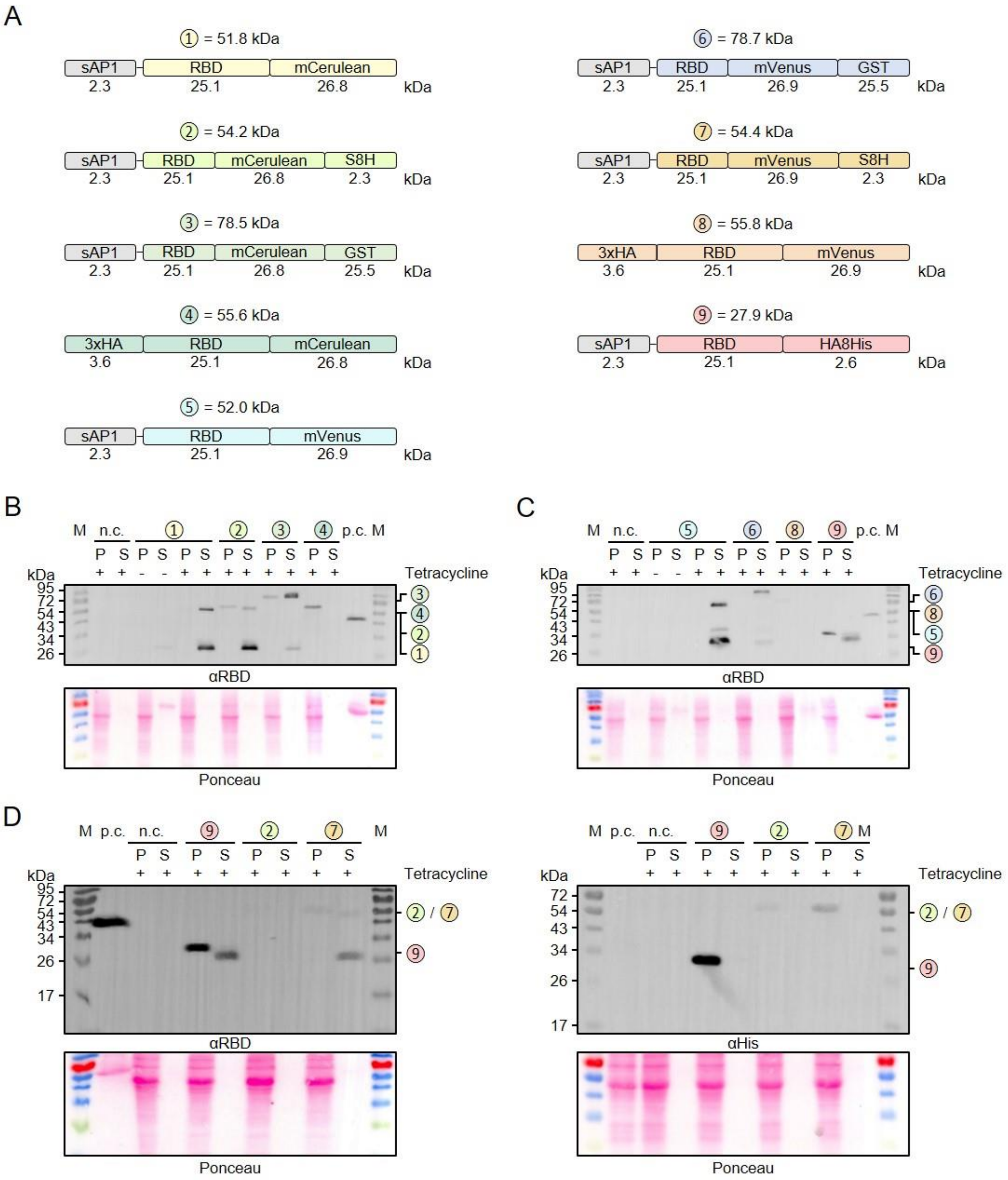

Supplementary Figure S1. Effect of C-terminal GST- or peptide tags on the stability of secreted RBD fusions with either mCerulean or mVenus. A) Schematic overview and expected mass of the secreted RBD fusion proteins. Fusion variants 4 and 8 with an N-terminal 3xHA tag instead of the N-terminal signal peptide served as cytosolic controls. Fusion variant 9 served as a RBD control without mCerulean or mVenus domain. S8H, Strep-8xHis tag; HA8His, HA-8xHis tag. **B)** Comparative Western blot analysis with an antibody against the RBD domain of RBD-mCerulean fusion proteins in the cell-containing pellet fraction (P) and supernatant fraction (S) following tetracycline induction of the according *L. tarentolae* liquid cultures. Recombinant RBD served as positive control (p.c.) and an induced culture without plasmid as negative control (n.c.). The calculated masses from panel A are indicated. **C)** Comparative Western blot analysis with an antibody against the RBD domain of RBD-mVenus variants. **D)** Comparative Western blot analysis with antibodies against the RBD domain or the His tag of C-terminally 8xHis-tagged fusion proteins.

Supplementary Figure S2

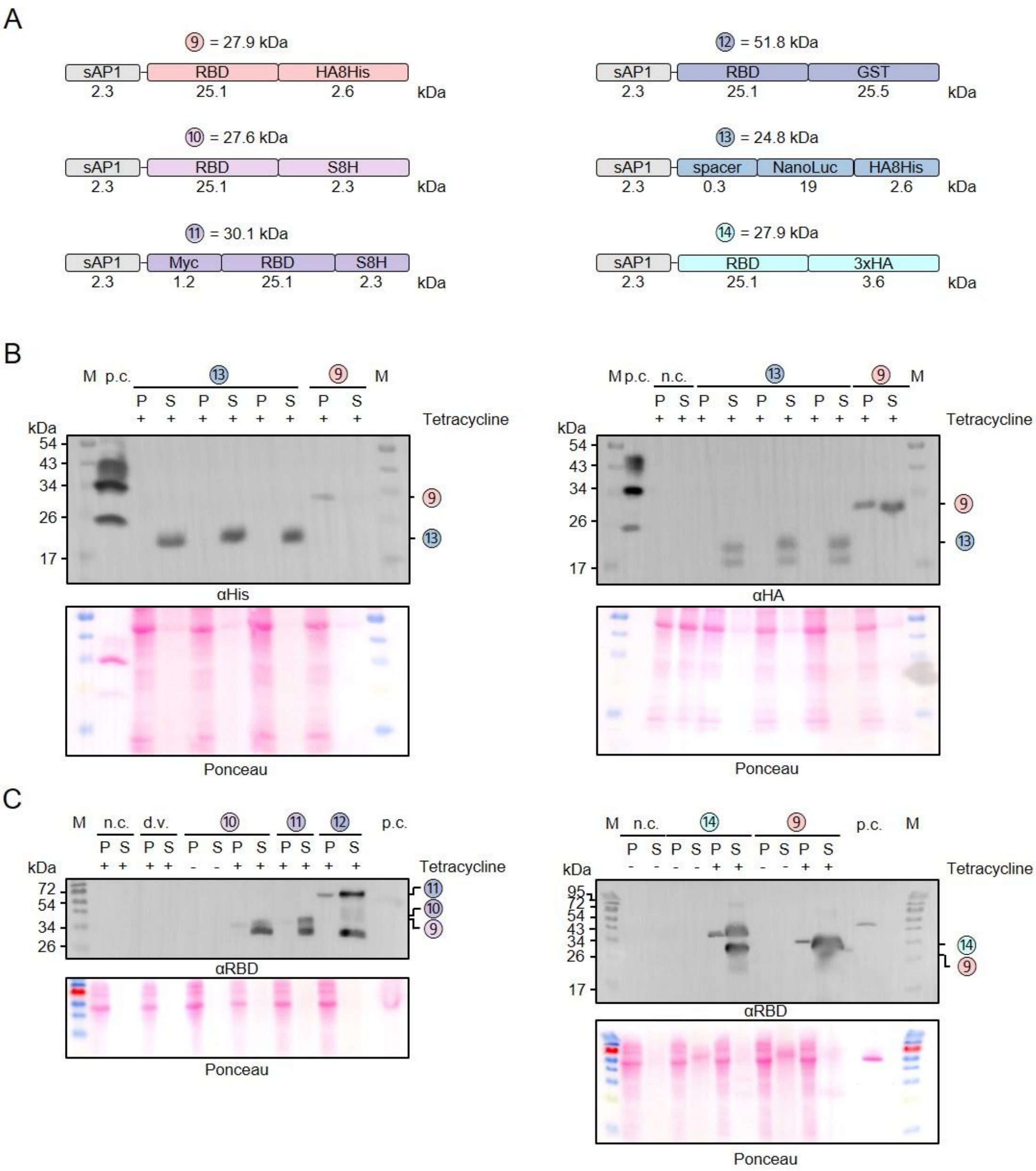

Supplementary Figure S2. Effect of different C-terminal tags on the stability of unfused secreted RBD. A) Schematic overview and expected sizes of the secreted RBD variants. Secreted NanoLuc was tested as an alternative protein. S8H, Strep-8xHis tag; HA8His, HA-8xHis tag. **B)** Comparative Western blot analysis with antibodies against the His and HA tags on RBD and NanoLuc in the cell-containing pellet fraction (P) and supernatant fraction (S) following tetracycline induction of the according *L. tarentolae* liquid cultures. Three different clones were analyzed for the NanoLuc construct. Recombinant RBD served as positive control (p.c.) and a mock-induced culture without plasmid as negative control (n.c.). The calculated masses from panel A are indicated. **C)** Comparative Western blot analysis with an antibody against RBD as in panel B. Induced cultures without plasmid (n.c.) or with domesticated empty vector (d.v.) served as negative controls and recombinant RBD served as a positive control (p.c.).

Supplementary Figure S3

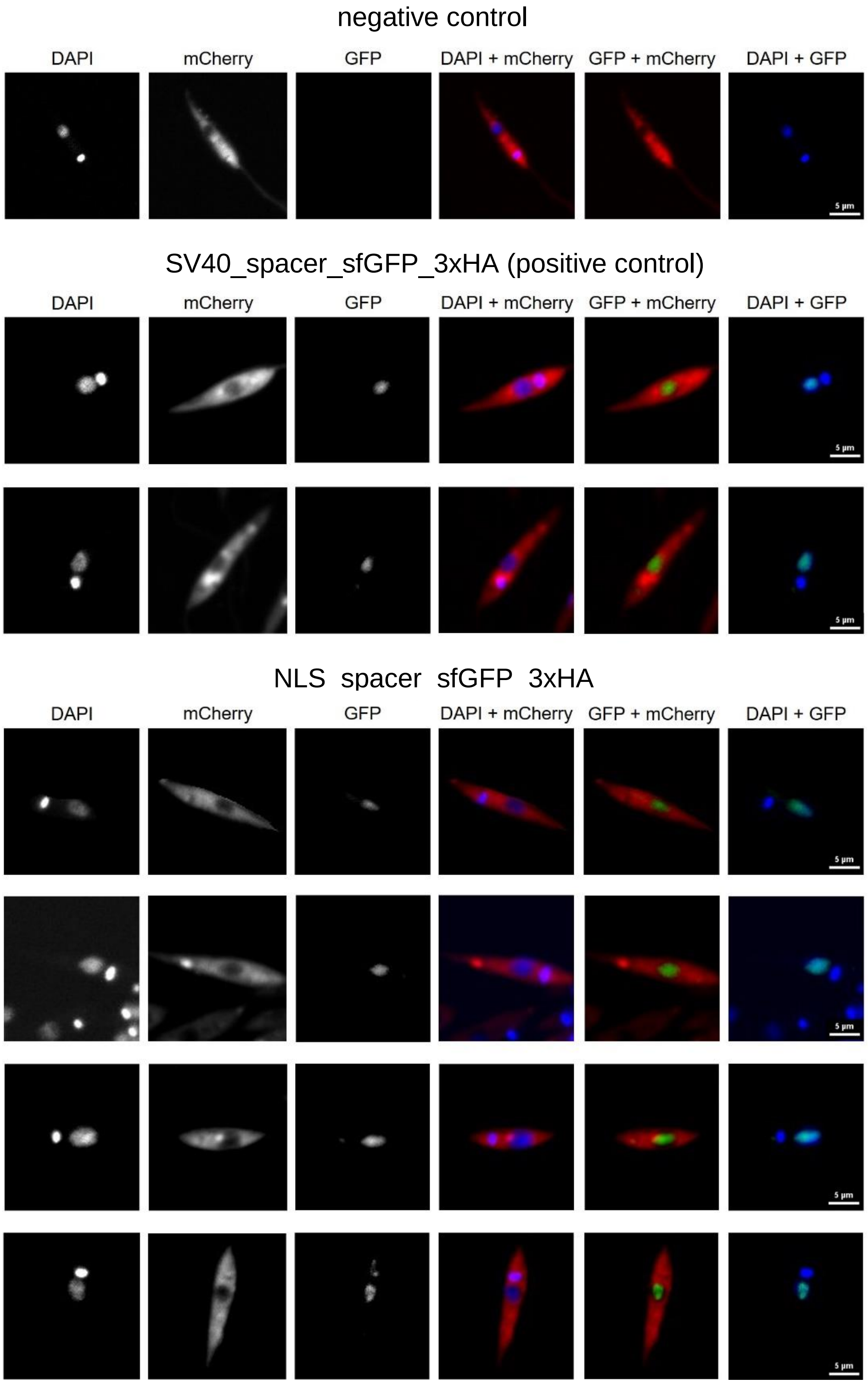

Supplementary Figure S3. Fluorescence microscopy confirms nuclear localization of NLS-tagged sfGFP. Nuclear and kinetoplast DNA of *L. tarentolae* promastigotes were stained with DAPI. A strain containing empty vector pLEXSY_I-blecherry3_dom_lacZ that encodes cytosolic mCherry served as a negative control (upper panels). Strains with two versions of plasmid-encoded NLS-tagged sfGFP are shown below. The monopartite NLS from construct SV40 served as a positive control (middle panels). The bipartite NLS from nucleoplasmin was analyzed for the cells that are shown at the bottom.

Supplementary Table 1. Oligonucleotides used for cloning, templates, and amplification products.

Oligonucleotide	Sequence	Target, product size
plex1-for	5’-AAAA**GGTCTC**TTGTCTCTCATGCTGCCCAATC-3’	pLEXSY_I-blecherry3,
plex2-rev	5’-AAAA**GGTCTC**AGGGACCGTGCGAGAGACGTATGC-3’	1718 nt
plex3-for	5’-AAAA**GGTCTC**TGCTCTCGCGGTATCATTGCAGC-3’	pLEXSY_I-blecherry3,
plex1-rev	5’-AAAA**GGTCTC**AGACAAAACGACCAAAGTTCCGAA-3’	1886 nt
plex2-for	5’-AAAA**GGTCTC**TTCCCGTTTATTTGATCATGTA-3’	pLEXSY_I-blecherry3,
plex400-rev	5’-AAAA**GGTCTC**AAATCTCCTTTCCTGCATC-3’	(475 nt)
plex400-for	5’-AAAA**GGTCTC**AGATTTACTGCAGGACGTCTA-3’	pLEXSY_I-blecherry3,
plexN-rev	5’-AAAA**GGTCTC**TGAGGTGATGTCCAACTTG-3’	1622 nt
plexN-for	5’-AAA**AGGTCTC**TCCTCCCACAACGAGGACT-3’	pLEXSY_I-blecherry3,
plex3-rev	5’-AAA**AGGTCTC**AGAGCCACGTCACCGGCTCCAGA-3’	2638 nt
BW-BglII-for	5’-AAAAAGATCTGCCATT**GAGACC**GTCACAGCTTGTC-3’	pICH47742,
BW-NotI-rev	5’-AAAAGCGGCCGCAAGCT**GAGACC**GCAGCTGGCACGA-3’	623 nt
SAP1-for	5’-AA**GAAGAC**AACCATGGCCTCGAGGCTCGTC-3’	*sAP1* in pLEXSY_I-
SAP1-rev	5’-TT**GAAGAC**ATCATTGCGCCAGCGTCGACCGACA-3’	blecherry3, 100 nt
3HA-B2-for	5’-AAAA**GAAGAC**GACCATGTACCCCTACGACG-3’	pCM0-100,
3HA-B2-rev	5’-AAAA**GAAGAC**ATCATTGCGGCAGCGTAGTCCG-3’	131 nt
MiMa-B2-for	5’-CCATGCTTCGTTCCGCAGTCCGCCTCGCTGGTAAGGACGTGCGTTTTGGCGAAGATGCA-3’	*mtHSP60*,
MiMa-B2-rev	5’-CATTTGCATCTTCGCCAAAACGCACGTCCTTACCAGCGAGGCGGACTGCGGAACGAAGC-3’	63 nt
Met-B2-for	5’-AA**GAAGAC**GTCCATGGCAATGCAGTCTTCTT-3’	None,
Met-B2-rev	5’-AA**GAAGAC**TGCATTGCCATGGACGTCTTCTT-3’	31 nt
NLS-B2-for	5’-CCATGAAAAGGCCGGCGGCCACGAAAAAGGCCGGCCAGGCAAAAAAGAAAAAGGG-3’	NLS nucleoplasmin,
NLS-B2-rev	5’-CATTCCCTTTTTCTTTTTTGCCTGGCCGGCCTTTTTCGTGGCCGCCGGCCTTTTC-3’	59 nt
Spacer-B3-for	5’-AATGAGCGGCGGCGGCGG-3’	None,
Spacer-B3-rev	5’-ACCTCCGCCGCCGCCGCT-3’	22 nt
RBD-B3-for	5’-AAAA**GAAGAC**CAAATGCGCGTGCAGCCGA-3’	pMBS857,
RBD-B3-rev	5’-AAAA**GAAGAC**CAACCTGCGAAGTTCACGCAC-3’	703 nt
RBD-B4-for	5’-AAAA**GAAGAC**CAAGGTCGCGTGCAGCCGA-3’	pMBS857,
RBD-B4-rev	5’-TTTT**GAAGAC**CACGAACCGAAGTTCACGC-3’	703 nt
mCe-for	5’-AAAA**GAAGAC**CAAATGATCGAGGGCAGGGTGA-3’	pCM0-046,
mCeIn-up	5’-TTTT**GAAGAC**CATTACCGTCGTCCTTGAAGAA-3’	354 nt
mCeIn-down	5’-AAAA**GAAGAC**AGGTAACTACAAGACCCG-3’	pCM0-046,
mCe-rev	5’-TTTT**GAAGAC**AACGAACCCTTCTCGAACTGC-3’	470 nt
mCe-B4-for	5’-AA**GAAGAC**GGAGGTGTGAGCAAGGGCGAGGA-3’	pMBS859,
mCe-B4-rev	5’-AA**GAAGAC**CTCGAACCCTTGTACAGCTCGTC-3’	744 nt
mCe-B5-for	5’-AA**GAAGAC**GGTTCGGTGAGCAAGGGCGAGGA-3’	pMBS859,
mCe-B5-rev	5’-AAA**GAAGAC**TGAAGCTTACTTGTACAGCTCGTC-3’	746 nt
mVe-for	5’-AAAA**GAAGAC**GTAATGGTGAGCAAGGGCGAG-3’	pCM0-066,
mVeIn-up	5’-AAAA**GAAGAC**GCCGTCGTCCTTGAAGAAGA-3’	338 nt
mVeIn-down	5’-AAAA**GAAGAC**AGGACGGTAACTACAAGACCC-3’	pCM0-066,
mVe-rev	5’-AAAA**GAAGAC**TACGAACCCTTGTACAGCTCGTC-3’	438 nt
mVe-B4-for	5’-AAAA**GAAGAC**GTAGGTGTGAGCAAGGGCGAG-3’	pMBS858,
mVe-B4-rev	5’-AA**GAAGAC**CTCGAACCCTTGTACAGCTCGT-3’	746 nt
mVe-B5-for	5’-AAAA**GAAGAC**GTTTCGGTGAGCAAGGGCGAG-3’	pMBS858,
mVe-B5-rev	5’-AAAA**GAAGAC**CGAAGCTTACTTGTACAGCTCGTC-3’	749 nt
8HisHA-B5-for	5’-TTCGCACCACCACCACCACCACCACCACCGCGGCAGCTACCCCTACGACGTGCCCGACTACGCTTAA-3’	None,
8HisHA-B5-rev	5’-AAGCTTAAGCGTAGTCGGGCACGTCGTAGGGGTAGCTGCCGCGGTGGTGGTGGTGGTGGTGGTGGTG-3’	71 nt
8HisSPHA-for	5’-AA**GAAGAC**AATTCGCACCACCACCACCACCACCACCACTCGCCCTCGCCCAGCCC	pMBS659,
8HisSPHA-rev	5’- TT**GAAGAC**AAAAGCTTAAGCGTAGTCGGGCACGT	208 nt
DGDL-B5-for	5’-TTCGGACGGCGACCTGTAG-3’	None,
DGDL-B5-rev	5’-AAGCCTACAGGTCGCCGTC-3’	23 nt
MDDL-B5-for	5’-TTCGATGGACGACCTGTAG-3’	None,
MDDL-B5-rev	5’-AAGCCTACAGGTCGTCCAT-3’	23 nt

BbsI (GAAGAC) and BsaI (GGTCTC) recognition sites are shown in bold, overhangs are underlined.
